# Supplementary material for: Metabolic Syndrome Prevention Potential of Tamarillo: Phytochemical Composition, Antioxidant Activity, and Enzyme Inhibition Before and After Digestion
Source: Foods. 2025 Apr 7;14(7):1282. doi: 10.3390/foods14071282 (PMC11988886; doi:10.3390/foods14071282)
Supplement: Supplementary file 1 [file foods-14-01282-s001.zip › foods-3559976-supplementary.pdf]

Supplementary materials

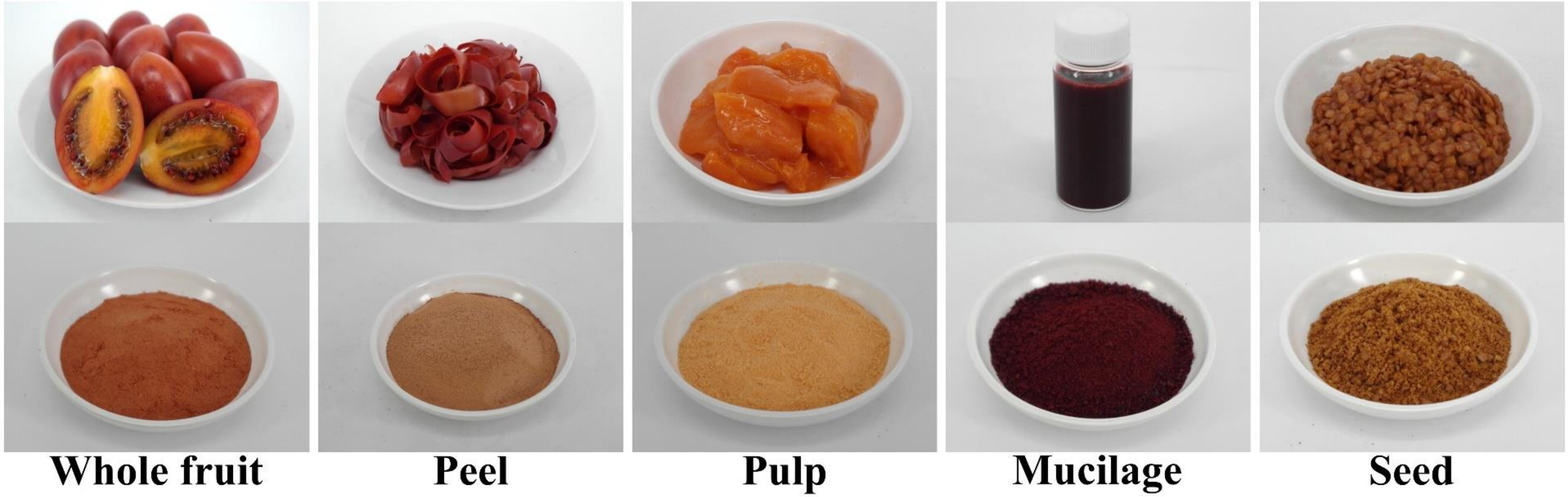

Figure S1. The appearance and color of fresh and freeze-dried powder of tamarillo whole fruit, peel, pulp, mucilage, and seed.

This image is reproduced from a previously published work by our research team: Chen, S.-Y.; Zhang, Q.-F.; Lin, S.-D. Nutritional and phytochemical composition of the red tamarillo grown in Taiwan. J. Food Compost. Anal. 2024, 131, 106258.

Table S1. The extraction yields of freeze-dried extracts.

Extraction method[1]	Extraction yield (%)[2]				
	Peel	Pulp	Mucilage	Seed	Whole
0E	26.72 ± 0.67 Ad[3]	65.16 ± 0.93 Ab	92.86 ± 0.48 Aa	17.81 ± 1.10 Ae	57.64 ± 0.98 Ac
25E	25.55 ± 0.35 Bd	63.36 ± 0.36 Bb	94.03 ± 0.23 Aa	16.83 ± 0.18 Ae	57.31 ± 0.70 Ac
50E	21.12 ± 0.32 Cd	62.09 ± 0.06 Bb	89.83 ± 1.32 Ba	14.88 ±< 0.01 Be	53.22 ± 0.51 Bc
75E	21.28 ± 0.27 Cd	62.38 ± 0.52 Bb	88.58 ± 0.45 Ca	6.29 ± 0.10 Ce	52.49 ± 0.14 Bc
95E	11.21 ± 0.12 Dd	41.17 ± 1.15 Cb	56.46 ± 0.12 Da	6.67 ± 0.42 Ce	33.68 ± 1.68 Cc

[1]0E, 25E, 50E, 75E and 95E: The freeze-dried extracts were prepared from freeze-dried tamarillo sample with water, 25%, 50%, 75% and 95% ethanol at 75 °C in a shaking water bath (150 rpm), respectively.

[2]Extraction yield (%) = (weight of freeze-dried extract/weight of sample) ×100%.

[3]Each value is expressed as mean ± standard deviation (n = 3). Means with different capital letters within a column differ significantly ($p < 0.05$). Means with different lowercase letters within a row differ significantly ($p < 0.05$).

A. Scavenging ability on DPPH B. Reducing power

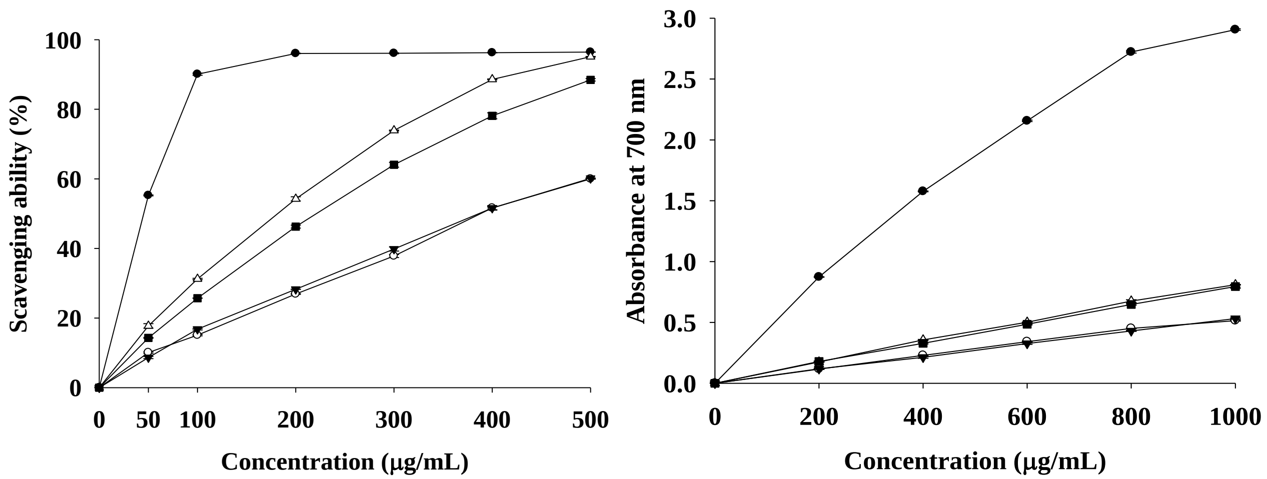

Figure S2. Scavenging ability on 2,2-diphenyl-1-picrylhydrazyl radicals and reducing power of freeze-dried extracts from freeze-dried tamarillo peel, pulp, mucilage, seed and whole fruit. Each value is expressed as mean ± standard deviation (n = 3). P95E (●), L95E (○), M95E (▼), S75E (△), W95E (■).

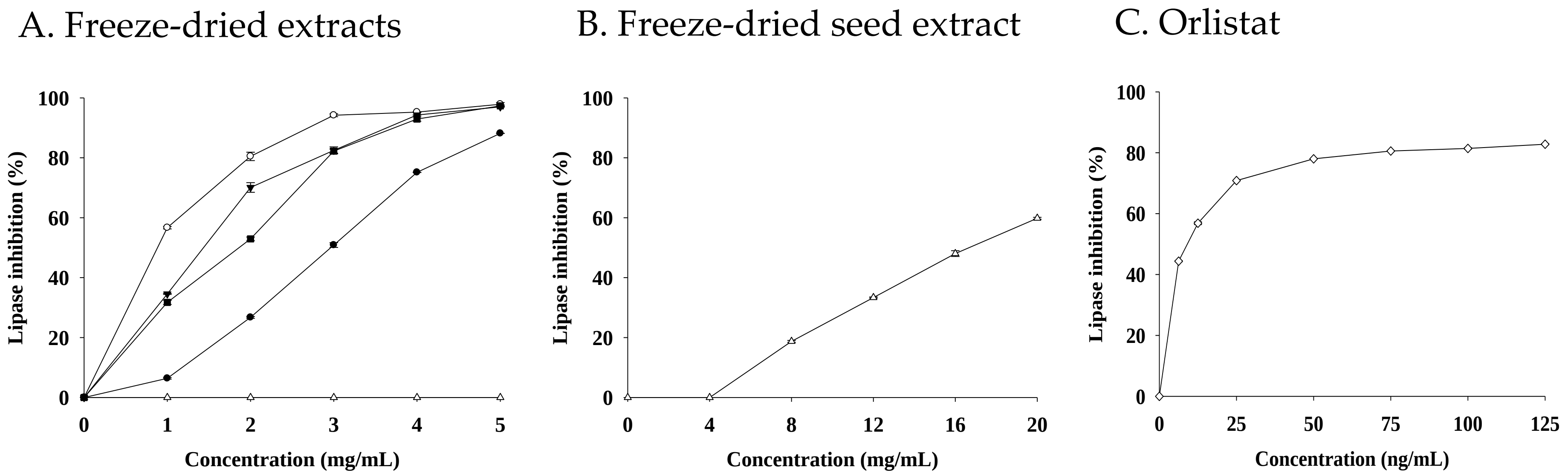

Figure S3. Inhibitory effects of freeze-dried extracts from freeze-dried tamarillo peel, pulp, mucilage, seed and whole fruit against pancreatic lipase. Each value is expressed as mean ± standard deviation (n = 3). A. P95E (●), L95E (○), M95E (▼), S75E (△), W95E (■). B. S75E (△). C. Orlistat (◊).

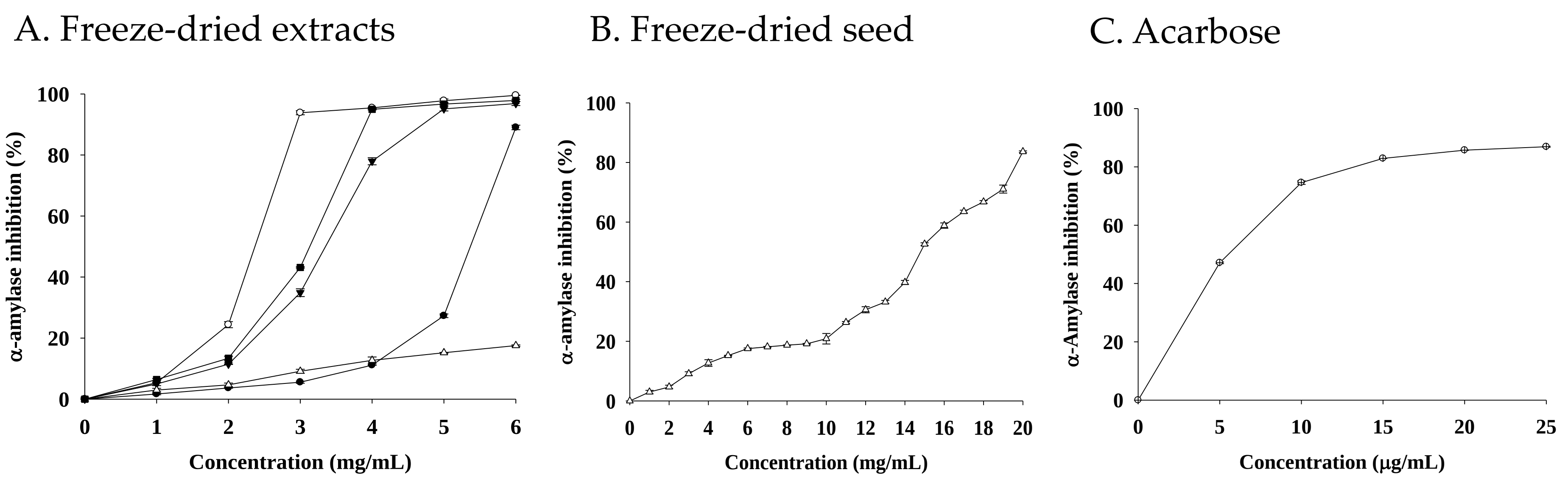

Figure S4. Inhibitory effects of freeze-dried extracts from freeze-dried tamarillo peel, pulp, mucilage, seed and whole fruit against α-amylase. Each value is expressed as mean ± standard deviation (n = 3). A. P95E (●), L95E (○), M95E (▼), S75E (△), W95E (■). B. S75E (△). C. Acarbose (♁).

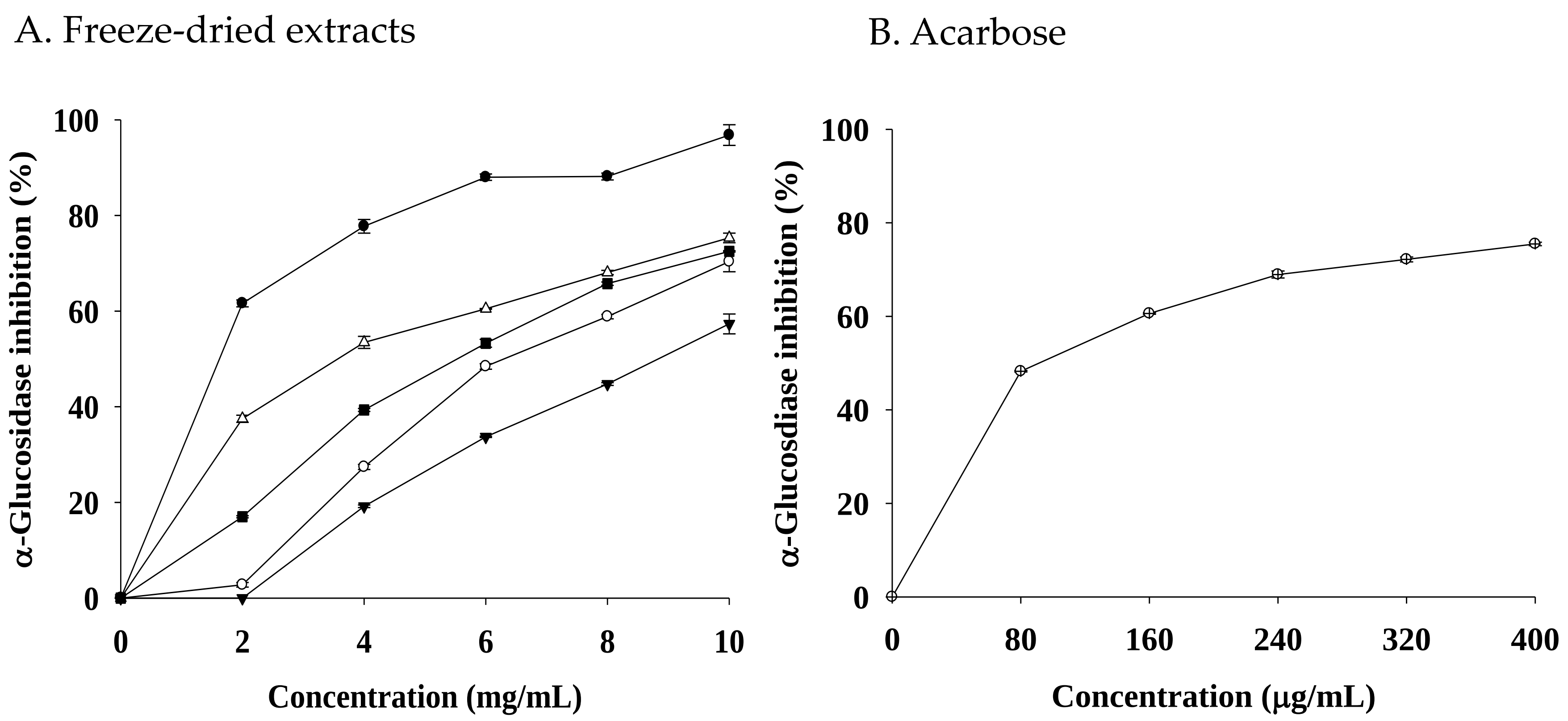

Figure S5. Inhibitory effects of freeze-dried extracts from freeze-dried tamarillo peel, pulp, mucilage, seed and whole fruit against α-glucosidase. Each value is expressed as mean ± standard deviation (n = 3). A. P95E (●), L95E (○), M95E (▼), S75E (△), W95E (■). B. Acarbose (♁).

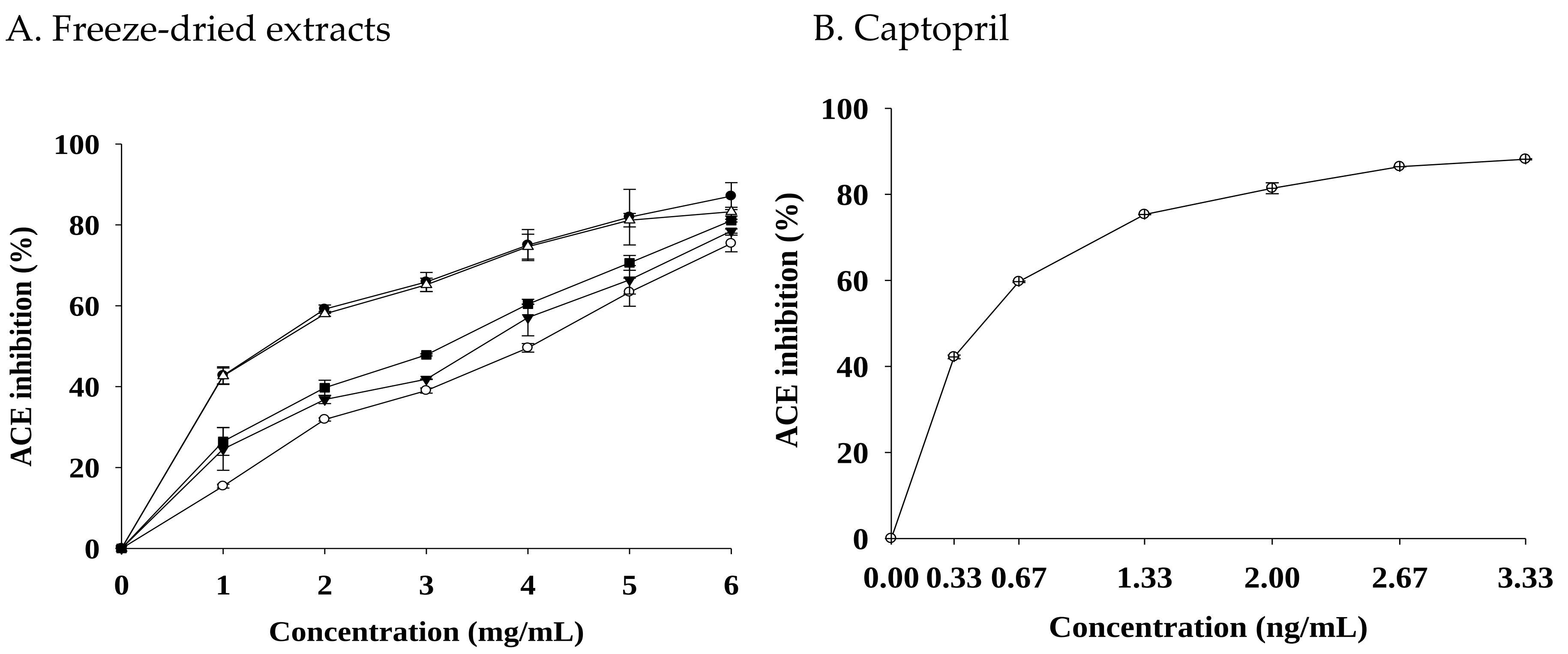

Figure S6. Inhibitory effects of freeze-dried extracts from freeze-dried tamarillo peel, pulp, mucilage, seed and whole fruit against angiotensin converting enzyme. Each value is expressed as mean ± standard deviation (n = 3). A. P95E (●), L95E (○), M95E (▼), S75E (△), W95E (■). B. Captopril (☆).
